# Supplementary material for: XRE Transcription Factors Conserved in Caulobacter and φCbK Modulate Adhesin Development and Phage Production
Source: bioRxiv. 2023 Aug 20:2023.08.20.554034. Preprint. [Version 1] doi: 10.1101/2023.08.20.554034 (PMC10462132; doi:10.1101/2023.08.20.554034)
Supplement: Supplement 6 [file NIHPP2023.08.20.554034v1-supplement-6.pdf]

bioRxiv preprint doi: https://doi.org/10.1101/2023.08.20.554034; this version posted August 20, 2023. The copyright holder for this preprint (which was not certified by peer review) is the author/funder, who has granted bioRxiv a license to display the preprint in perpetuity. It is made available under aCC-BY 4.0 International license.

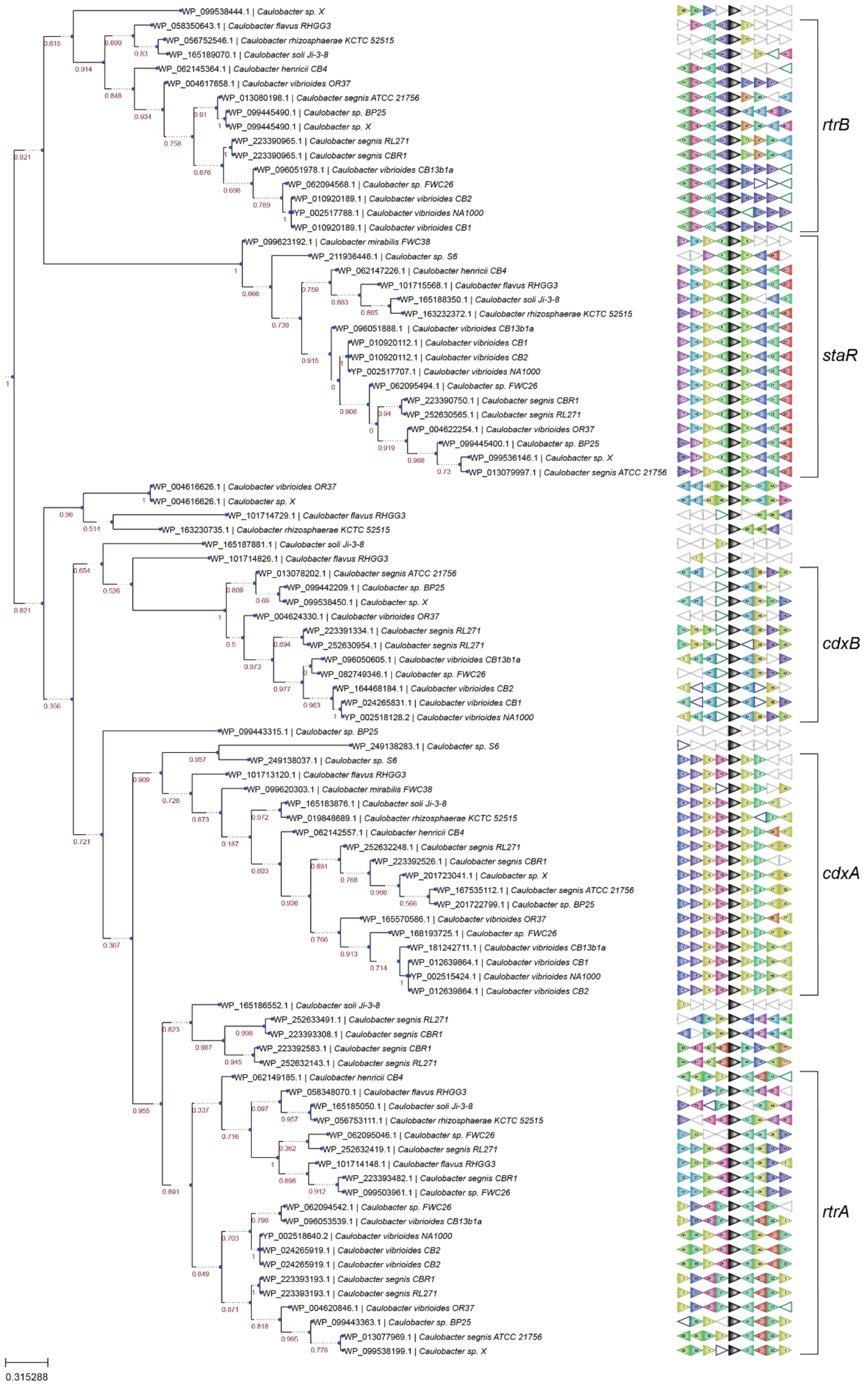

bioRxiv preprint doi: https://doi.org/10.1101/2023.08.20.554034; this version posted August 20, 2023. The copyright holder for this preprint (which was not certified by peer review) is the author/funder, who has granted bioRxiv a license to display the preprint in perpetuity. It is made available under aCC-BY 4.0 International license.

Figure S1. XRE transcription factor paralogs have conserved genomic neighborhoods in the Caulobacter species. Genomic neighborhood analysis of XRE transcription factor paralogs. Phylogenetic tree based on XRE transcription factor sequences (left) and genomic neighborhood surrounding those genes (right). Protein sequences were retrieved using the protein accession numbers and associated GCF assembly IDs for proteins from bins GC_0003, GC_0408, and GC_2778 in the pangenome analysis (Figure 2A) and analyzed with the webFLaGs server (https://server.atkinson-lab.com/webflags) [73]. Numbers on the phylogenetic tree indicate bootstrap values. XRE homologs are colored black, orthologous genes are colored and numbered identically, non-conserved genes are uncolored and outlined in grey, pseudogenes are uncolored and outlined in blue, and non-coding RNA genes are uncolored and outlined in green.

bioRxiv preprint doi: https://doi.org/10.1101/2023.08.20.554034; this version posted August 20, 2023. The copyright holder for this preprint (which was not certified by peer review) is the author/funder, who has granted bioRxiv a license to display the preprint in perpetuity. It is made available under aCC-BY 4.0 International license.

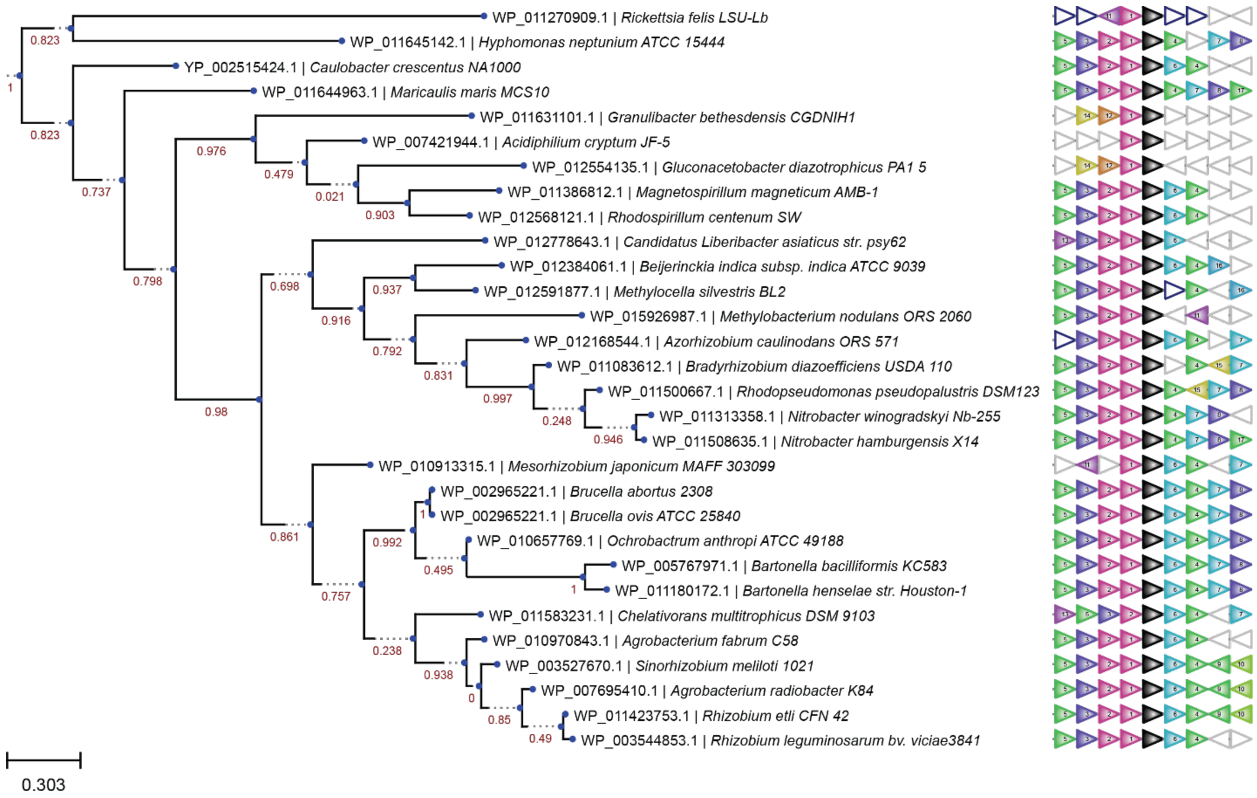

Figure S2. *cdxA* homologs and surrounding genomics neighborhood are conserved across Alphaproteobacteria. Phylogenetic tree based on XRE transcription factor sequences (left) and genomic neighborhood surrounding those genes (right). Protein accession numbers (were modified from [25]) and associated GCF assembly IDs were analyzed with the webFLaGs server (https://server.atkinson-lab.com/webflags) [73]. Numbers on the phylogenetic tree indicate bootstrap values. *cdxA* homologs are colored black, orthologous genes are colored and numbered identically, non-conserved genes are uncolored and outlined in grey, pseudogenes are uncolored and outlined in blue, and non-coding RNA genes are uncolored and outlined in green.

bioRxiv preprint doi: https://doi.org/10.1101/2023.08.20.554034; this version posted August 20, 2023. The copyright holder for this preprint (which was not certified by peer review) is the author/funder, who has granted bioRxiv a license to display the preprint in perpetuity. It is made available under aCC-BY 4.0 International license.

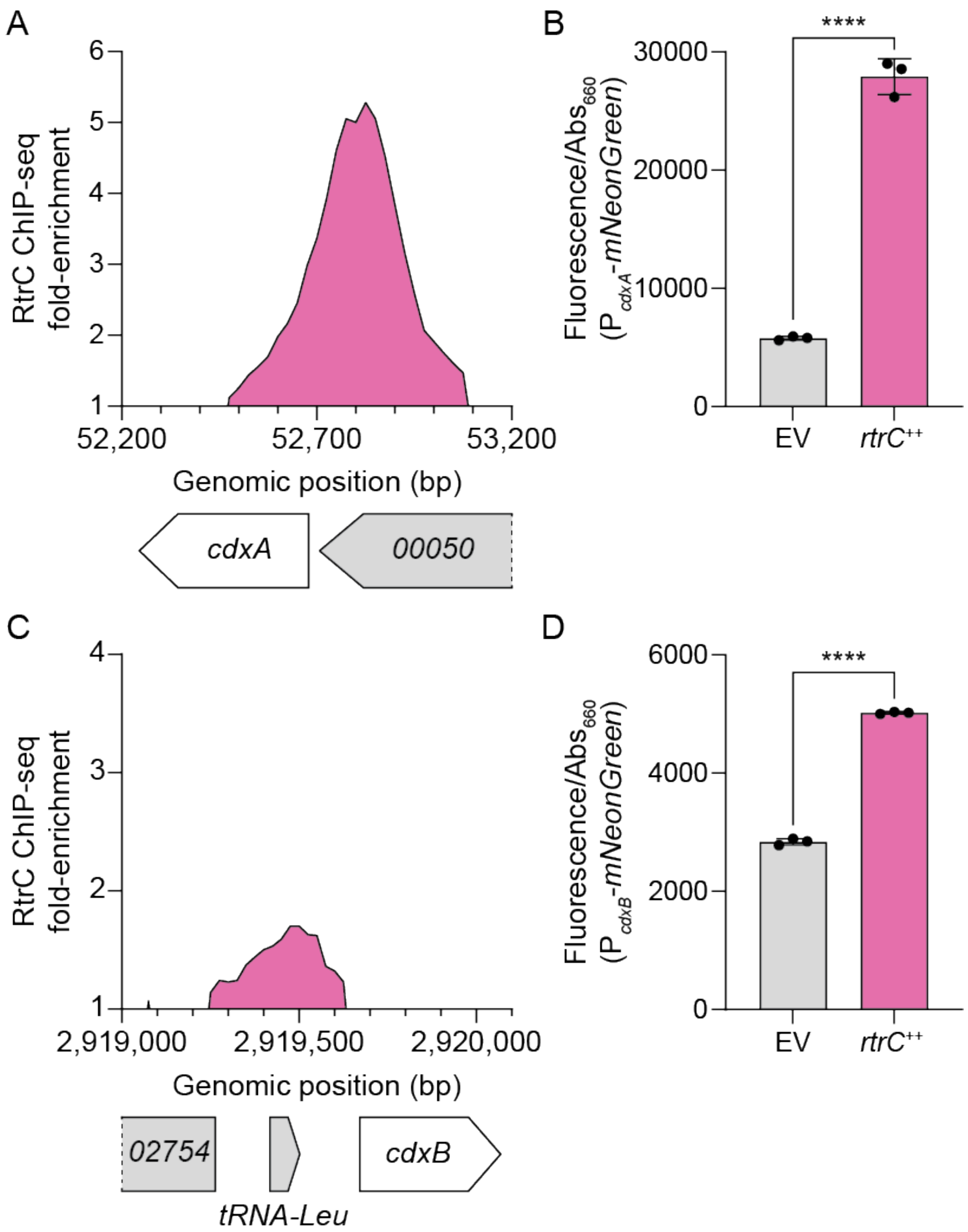

Figure S3. RtrC activates expression of *cdxA* and *cdxB*. A & C) RtrC binds the *cdxA* and *cdxB* promoter *in vivo*. ChIP-seq profile from pulldowns of 3xFLAG-tagged protein are shown. Lines indicate the fold-enrichment from pulldowns compared to an input control. Genomic position and relative position of genes are indicated. Data are in 25 bp bins and are the mean of three biological replicates. **B & D)** *cdxA* and *cdxB* expression using a P_{cdxA}- or P_{cdxB}-*mNeonGreen* reporter. Fluorescence was measured in either a wild type background containing either an empty vector (EV) or *rtrC* overexpression (++) vector. Fluorescence was normalized to cell density. Data are the mean and error bars are the standard deviation of three biological replicates. Statistical significance was determined by multiple unpaired t-test using the Holm-Šídák method to correct for multiple comparisons (p-value $\leq$ 0.0001,****).

bioRxiv preprint doi: https://doi.org/10.1101/2023.08.20.554034; this version posted August 20, 2023. The copyright holder for this preprint (which was not certified by peer review) is the author/funder, who has granted bioRxiv a license to display the preprint in perpetuity. It is made available under aCC-BY 4.0 International license.

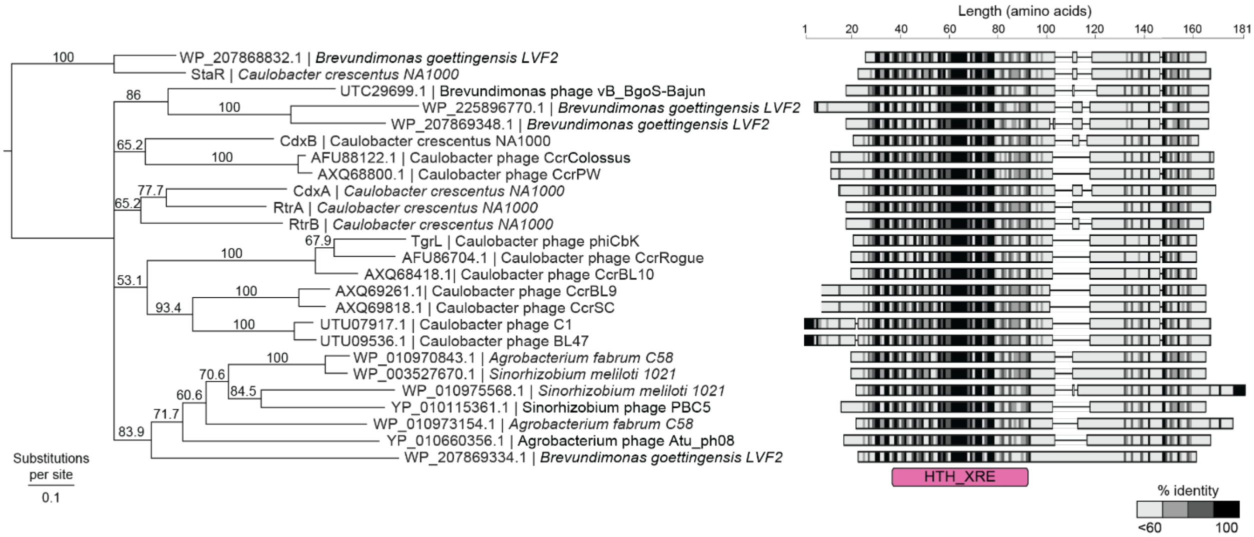

Figure S4. XRE homologs are encoded by Alphaproteobacterial phage. Phylogenetic tree (left) and multiple sequence alignment (right) of XRE homologs from different *Alphaproteobacteria* and Alphaproteobacterial phage. Numbers above branches indicate percent bootstrap support and branch length corresponds to substitutions per site. Protein accession numbers and organism are displayed next to corresponding branches. Alignments (right) match the order in the phylogenetic tree (left). For alignments, horizontal lines indicate gaps. Pink rectangle indicates the location of the HTH_XRE domain.

bioRxiv preprint doi: https://doi.org/10.1101/2023.08.20.554034; this version posted August 20, 2023. The copyright holder for this preprint (which was not certified by peer review) is the author/funder, who has granted bioRxiv a license to display the preprint in perpetuity. It is made available under aCC-BY 4.0 International license.

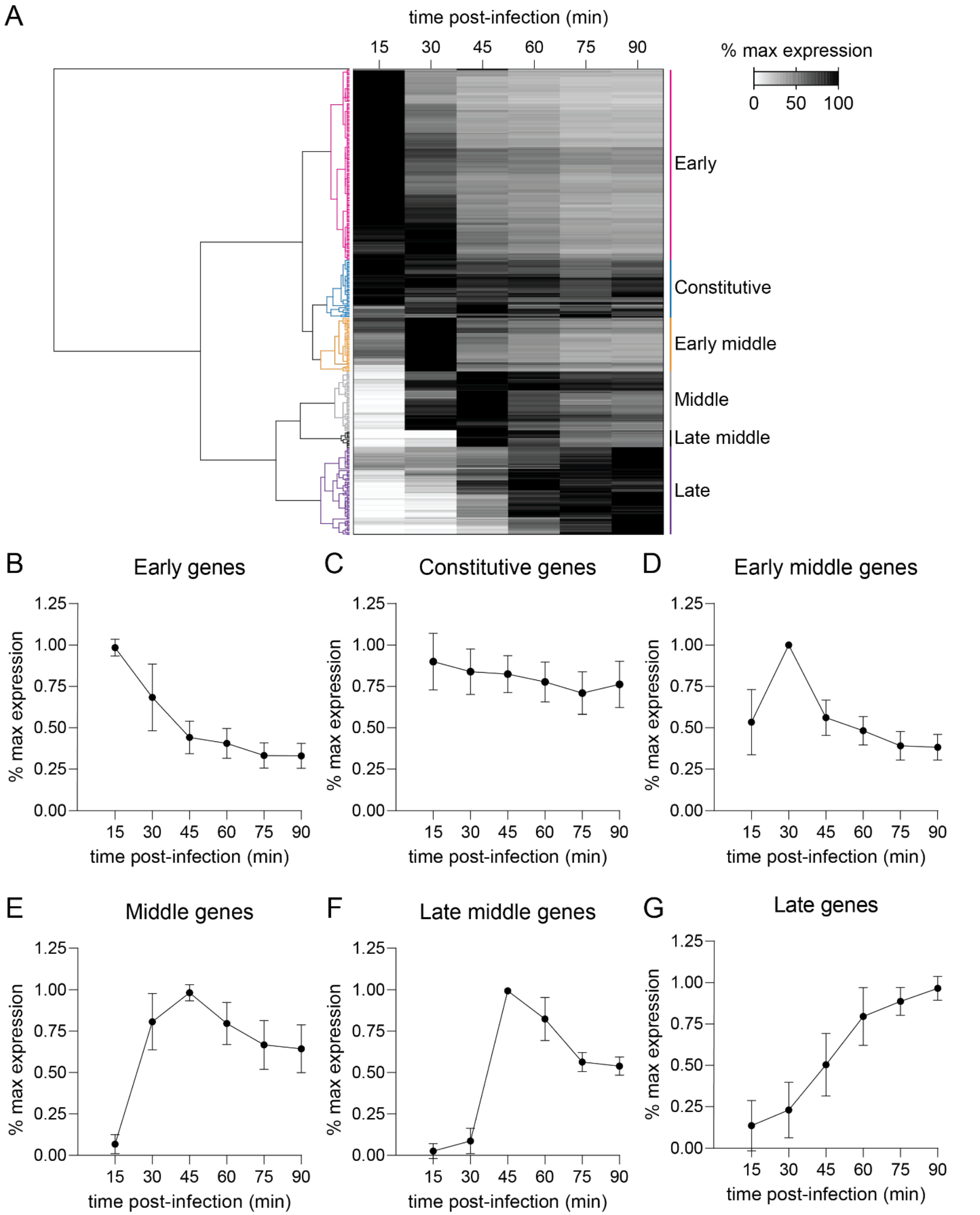

Figure S5. φCbK genes are expressed in distinct temporal patterns throughout infection. A) Hierarchical clustering of φCbK gene expression during infection of *C. crescentus*. Relative values (i.e. % max expression) were calculated by normalizing transcript levels at a time point to the maximum transcript levels for that gene over the infection time course. Relative gene

bioRxiv preprint doi: https://doi.org/10.1101/2023.08.20.554034; this version posted August 20, 2023. The copyright holder for this preprint (which was not certified by peer review) is the author/funder, who has granted bioRxiv a license to display the preprint in perpetuity. It is made available under aCC-BY 4.0 International license.

expression was hierarchically clustered using Cluster 3.0 [79] and plotted as a heatmap. Rows correspond to φCbK genes and clusters are colored and labeled. Data are the mean of three biological replicates. **B-G)** Relative gene expression of clusters from hierarchical clustering. Data are the mean relative expression of all genes within the indicated cluster and error bars are the associated standard deviations. Wild type cells were infected during logarithmic growth phase in complex medium (PYE) at 10 multiplicity of infection (MOI).

bioRxiv preprint doi: https://doi.org/10.1101/2023.08.20.554034; this version posted August 20, 2023. The copyright holder for this preprint (which was not certified by peer review) is the author/funder, who has granted bioRxiv a license to display the preprint in perpetuity. It is made available under aCC-BY 4.0 International license.

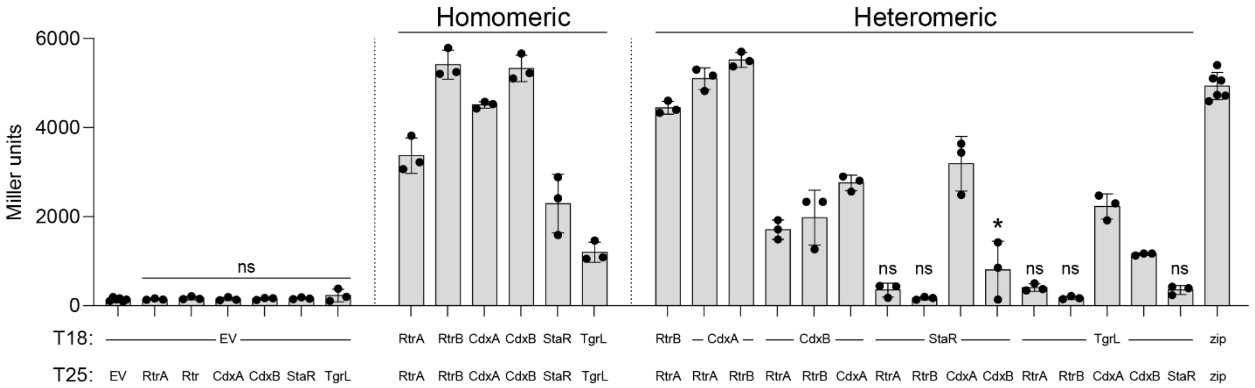

Figure S6. *C. crescentus* and φCbK XRE proteins form homo- and heteromeric interactions. Heatmap showing the interaction between RtrA, RtrB, CdxA, CdxB, StaR, and φCbK TgrL based on bacterial two-hybrid (BTH) assays. Proteins were fused to split adenylate cyclase fragments (T18c and T25) and co-expressed. Interactions between the fused proteins reconstitutes adenylate cyclase, promoting expression of a *lacZ* reporter. Empty vector (EV) are the negative control and Zip is the positive control. β-galactosidase activity was measured, and Miller units were calculated. Data are the mean and error bars are the standard deviation of at least three biological replicates. Statistical significance was determined by one-way ANOVA compared to the EV only control followed by Dunnett's multiple comparison. Non-significant columns are indicated with (ns). Columns with p-value $\leq$ 0.05 were indicated with (*). All other columns had p-values $\leq$ 0.0001.
